# Prevalence of udder pathogens in milk samples from Norwegian dairy cows recorded in a national database in 2019 and 2020

**DOI:** 10.1186/s13028-023-00681-2

**Published:** 2023-06-01

**Authors:** Marit Smistad, Haakon Christopher Bakka, Liv Sølverød, Hannah Joan Jørgensen, Cecilia Wolff

**Affiliations:** 1 TINE Mastitis Laboratory, P.O. Box 2038, 6402 Molde, Norway; 2 Norwegian Veterinary Institute, P.O. Box 64, 1431 Ås, Norway

**Keywords:** Antimicrobial resistance, Bovine udder health, Intramammary infection, Mastitis, Milking system, Staphylococci, Streptococci, Udder pathogens

## Abstract

**Background:**

Identification of aetiological agents of mastitis in dairy cattle is important for herd management of udder health. In Norway, results from mastitis diagnostics are systematically recorded in a central database, so that the dairy industry can follow trends in the recorded frequency of udder pathogens and antimicrobial resistance patterns at national level. However, bacteriological testing of milk samples is based on voluntary sampling, and data are therefore subject to some bias. The aim of this study was to examine the prevalence of udder pathogens in Norwegian dairy cows by analysing data from the national routine mastitis diagnostics and to explore how routines for sampling and diagnostic interpretations may affect the apparent prevalence of different bacterial pathogens. We also assessed associations between udder pathogen findings and the barn- and milking systems of the herds.

**Results:**

The most frequently detected major udder pathogens among all milk samples submitted for bacterial culture (n = 36,431) were *Staphylococcus aureus* (24.5%), *Streptococcus dysgalactiae* (13.3%) and *Streptococcus uberis* (9.0%). In the subset of samples from clinical mastitis (n = 7598); *Escherichia coli* (14.5%) was the second most frequently detected pathogen following *S. aureus* (27.1%). *Staphylococcus epidermidis* (10.0%), *Corynebacterium bovis* (9.4%), and *Staphylococcus chromogenes* (6.0%) dominated among the minor udder pathogens. Non-aureus staphylococci as a group, identified in 39% of the sampling events, was the most frequently identified udder pathogen in Norway. By using different definitions of cow-level bacterial diagnoses, the distribution of minor udder pathogens changed.

Several udder pathogens were associated with the barn- and milking system but the associations were reduced in strength when data were analysed from farms with a comparable herd size. *S. aureus* was associated with tiestall housing, *E. coli* and *S. dysgalactiae* were associated with freestall housing, and *S. epidermidis* was associated with automatic milking systems.

Only 2.5% of the 10,675 tested *S. aureus* isolates were resistant to benzylpenicillin. Among the 2153 tested non-aureus staphylococci, altogether 34% were resistant to benzylpenicillin.

**Conclusions:**

This study presents the recorded prevalence of udder pathogens in Norway over a two-year period and assesses the possible impact of the sampling strategies, diagnostic methods and diagnostic criteria utilized in Norway, as well as associations with different housing and milking systems. The national database with records of results from routine mastitis diagnostics in Norway provides valuable information about the aetiology of bovine mastitis at population level and can reveal shifts in the distribution and occurrence of udder pathogens.

**Supplementary Information:**

The online version contains supplementary material available at 10.1186/s13028-023-00681-2.

## Background

Mastitis is the most common reason for antimicrobial treatment of dairy cows in modern dairy production and remains a main challenge for the dairy industry because of its negative impact on milk quality, animal welfare, and farm economy [1, 2].

Information about the distribution and frequency of different bacterial pathogens associated with intramammary infections (IMI) is crucial for developing herd-level control strategies.

In Norway, the dairy co-operative TINE SA operates a mastitis laboratory that presently performs all bacteriological analyses of milk samples. Veterinary practitioners rarely carry out bacterial culturing themselves. The laboratory records are entered into a central database, the Norwegian Dairy Herd Recording System (NDHRS). The database also includes cow- and herd level recordings on production, disease and treatments, and reproductive events. This benefits farmers, dairy advisors, and veterinarians by providing the basis for decision support and pathogen specific preventive measures through digital tools (www.medlem.tine.no) that combine results from mastitis diagnostics and other herd recordings [3]. Another important benefit of a central database is the possibility to use the data for udder health surveillance at national level.

**Fig. 1:**
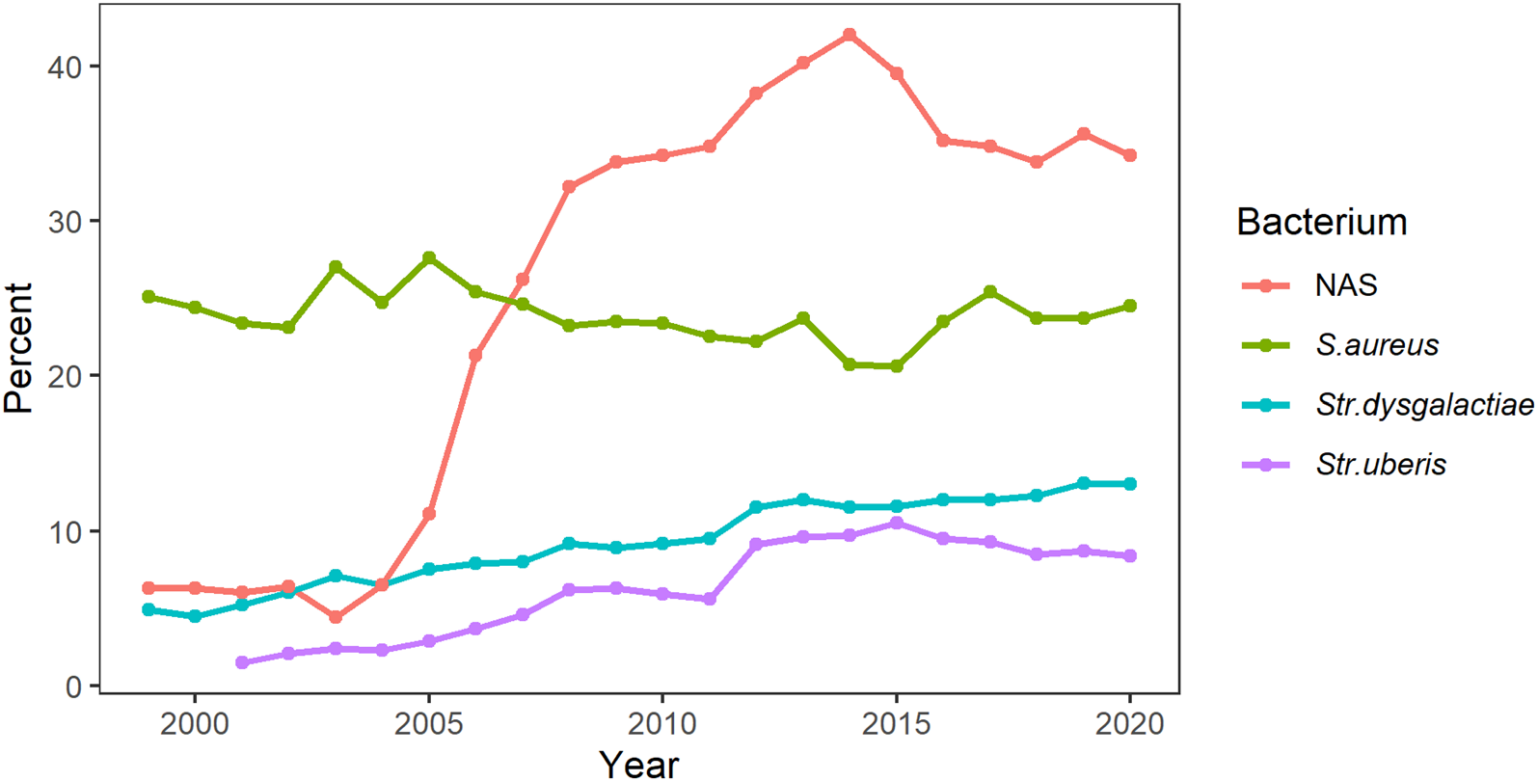
**Distribution of selected udder pathogens in milk samples from Norwegian dairy cows between 2000 and 2020.** Summary of bacterial findings reported by the national mastitis laboratories (TINE Mastitis Laboratory and the Norwegian Veterinary Institute until 2018) to the Norwegian dairy herd recording system between 2000 and 2020 [6]. Number of sampling events (sets of quarter milk samples) per year ranged from 6563–18,044. Data are presented as the percentage of cows receiving the diagnosis in at least one quarter, meaning that each cow can have up to four diagnoses per sampling event. The marked increase of non-aureus staphylococci (NAS) after 2005 is explained by altered diagnostic criteria for defining an intramammary infection caused by NAS

Bacterial findings in milk samples submitted to the mastitis laboratories have been presented in annual summaries (Fig. 1), but these do not account for clustering at cow and herd level, reasons for sampling, or the relevance of detecting multiple bacterial species in a cow.

The Norwegian dairy industry has undergone significant structural changes during the last two decades that may influence the udder pathogen panorama of dairy cows. The number of dairy farms decreased from more than 20,000 in 2000 to less than 7,000 in 2022 while the number of dairy cows only decreased from approximately 300,000 to 200,000 in the same period [4]. An ongoing regulatory enforced transition to freestall housing has contributed to many farmers investing in automatic milking systems (AMS), which in turn contributes to larger herds, higher production, and changes in management [5]. Today, more than 50% of the milk produced in Norway comes from farms with AMS [6].

In Norway, increasing herd size is associated with fewer mastitis treatments per cow, but slightly higher bulk somatic cell count [6]. New management systems may lead to changes in the dairy cow environment as well as altered routines for milk sampling and treatment, and may therefore influence the prevalence of udder pathogens.

The introduction of new diagnostic methods may alter the spectrum of bacterial species identified. In Norway, a commercial qPCR (Mastit 4, DNA Diagnostic) has been used as a supplement to bacterial culture in the mastitis diagnostics since 2013. With respect to culturing, Matrix-Assisted Laser Desorption Ionization Time of Flight (MALDI TOF) was introduced to the mastitis diagnostics in Norway in 2016, which offers rapid identification of bacterial species that were previously grouped together, like the non-aureus staphylococci (NAS).

The apparent (recorded) prevalence of udder pathogens is affected by diagnostic criteria, laboratory methods, and sampling practices, while the true prevalence of udder pathogens is influenced by, among other things, alterations in management and the dairy cow environment.

The primary aim of this study was to analyse and present results from two recent years of mastitis diagnostics in Norwegian dairy cows with the laboratory diagnostic methods and diagnostic criteria applied in 2020. Since the Norwegian milk production is transitioning from tiestall to freestall housing of cattle, often with AMS, a secondary aim was to investigate whether the distribution of udder pathogens is associated with the barn- and milking system.

## Methods

### Data registry

For this study we used data extracted from the NDHRS, which included both herd data and results from routine milk samples recorded in the period from January 1st 2019 to December 31st 2020. The bacteriological results in this database were recorded by the accredited mastitis laboratory owned by the largest Norwegian dairy cooperative (TINE SA). It analyses samples from dairy farms across the country and is currently the only laboratory performing mastitis diagnostics in Norway. In addition to bacteriological results from analyses of milk samples, the extracted dataset also included herd data from each of the farms from which at least one milk sample was submitted in the study period.

In 2020, the average herd size in Norway was 29 cows, and the average annual milk yield per cow was 8204 kg [4]. Norwegian red, a breed that is optimized for both beef and milk production, accounts for more than 90% of the national herd [6].

In the following text we describe routines for sampling of Norwegian dairy cows, as well as methods for bacteriological analyses of samples, in order to provide background information about the dataset extracted from the NDHRS for this study.

### Background about the dataset: routines for milk sampling and treatment of mastitis in Norwegian dairy cattle

Comprehensive national recommendations regarding sampling routines and antimicrobial treatment of bovine mastitis are available in Norway [7]. They state that antimicrobial treatments should be reserved for moderate/severe mastitis during lactation, and to treat subclinical mastitis caused by *Staphylococcus aureus, Streptococcus dysgalactiae, Streptococcus uberis* or *Streptococcus agalactiae* at dry-off. Benzylpenicillin procaine is the first choice for mastitis treatment in Norway [7]. Only veterinarians can prescribe antimicrobial treatments, and the reporting of veterinary treatments is mandatory [8, 9].

Quarter milk samples for bacterial culture are collected by the farmer or the veterinarian, and all four udder quarters are routinely sampled. The recommendation is to submit milk samples for bacterial culture before commencing antimicrobial treatment, and before dry-off if the cow had a geometric mean somatic cell count (SCC) of more than 100,000 cells/mL at the three last milk recordings. At dry-off, the farmers may choose to submit composite cow samples for the Mastit 4 M4A qPCR (DNA Diagnostics, Risskov, Denmark), which detects gene segments specific to *S. aureus, S. dysgalactiae, S. uberis* and *S. agalactiae*. Finally, farmers may also request milk samples when purchasing lactating cows, and to follow up individual cows after mastitis treatment.

Upon submission of milk samples to the laboratory, the reason for sampling is indicated in the requisition form, which provides altogether 15 alternatives, including, e.g., “clinical mastitis”, “high somatic cell count”, “control after mastitis treatment”, “*S. agalactiae*-control”, and “control before dry-off”. Milk samples are transported cooled with the milk truck from the farm to the laboratory, which usually takes between one and three days.

### Background about the dataset: laboratory analyses

In the routine mastitis diagnostics at the TINE mastitis laboratory, bacterial culture of milk samples is performed according to standard procedures [10]. Briefly, 0.01 mL of milk from each quarter are spread on washed 5% cattle blood agar plates with esculin and incubated at 37 °C. Plates are read at 24 h and 48 h.

Bacterial findings are reported if bacteria grow in pure culture and with five or more colonies (500 cfu/mL). With the exception of *Corynebacterium bovis* with typical colony morphology and *S. aureus* with typical colony morphology and betatoxic haemolysis, colonies are identified with MALDI TOF (Bruker Daltonics, Bremen, Germany). Non-aureus staphylococci are reported at species-level for *Staphylococcus epidermidis*, *Staphylococcus chromogenes, Staphylococcus simulans, Staphylococcus warneri, Staphylococcus haemolyticus* and *Staphylococcus caprae.* Otherwise, they are grouped as NAS.

Samples from cows that are treated with antimicrobials the week before sampling (as stated in the requisition form), are tested for inhibitory substances.

Since benzylpenicillin procaine is used in the majority of treatments of Norwegian dairy cows, *S. aureus* (all reasons for sampling) and NAS (from clinical mastitis primarily) are routinely tested for betalactamase production by the cloverleaf assay [11]. Streptococci are considered sensitive to benzylpenicillin and are not routinely tested. Penicillin-resistant *S. aureus* and *Enterobacteriaceae* from clinical mastitis are tested by disc diffusion for amoxicillin – clavulanic acid, ampicillin, cefoxitin (*S. aureus*) and trimethoprim sulpha (*Enterobacteriaceae*) (results not included in this study).

For qPCR-analysis, DNA-extraction and analysis are performed according to the manufacturer’s instructions (Mastit 4 qPCR, DNA Diagnostics, Risskov, Denmark).

### Data analysis

#### Data preparation

Milk sample results and herd data for the period 1.1.2019 to 31.12.2020 were retrieved from the NDHRS. A sampling event was defined as: *quarter samples taken on the same day from one cow*. When a cow had multiple sampling events yielding the same pathogen during the study period, only the first sampling event was included. The reasons for sampling “high somatic cell count” and “control before dry-off” were combined to one category termed “subclinical mastitis”. The samples from mild, moderate, and severe clinical mastitis were combined to the category termed “clinical mastitis”. Samples were excluded if the reason for sampling was “control after mastitis treatment”, if inhibitory substances were detected, or if the previous sampling event was less than four days earlier.

### Definition of cow-level bacterial diagnosis

The set of quarter milk samples obtained at a sampling event is usually from a mixture of clinically healthy and diseased (subclinical or clinical mastitis) quarters of the cow, but information about which quarter that was the reason for the sampling was not available in the NDHRS. Furthermore, the majority of samples came from cows with subclinical mastitis that are selected for sampling based on SCC at cow-level. Therefore, different definitions for aggregating the sampling event (i.e., the set of quarter-level diagnoses) to cow-level bacterial diagnoses were applied when analysing the dataset:

- In “definition 1” the bacterial finding of each quarter was included independently of the other quarter samples, so that cows with different bacterial findings from different quarters at the same sampling event contributed equally to the reported prevalence.
- In “definition 2” we assumed that some selected major udder pathogens were the primary reason for sampling. Cows with one quarter positive for *S. aureus*, *S. dysgalactiae*, *S. uberis*, *Escherichia coli*, *Trueperella pyogenes* or *S. agalactiae* (“major” pathogens) were assigned to this pathogen as the cow-level diagnosis. If the cow had two or more major pathogens (in different quarters) at the same sampling event, the diagnosis was set as “mixed major” IMI. Cows were given a cow-level diagnosis for *S. epidermidis, S. chromogenes, S. simulans, S. haemolyticus* or *C. bovis* (“minor” pathogens) if they had no major udder pathogen at the same sampling event. If two different minor pathogens were present at the same sampling event, the diagnosis was set as “mixed minor” IMI.
- In “definition 3”, a data subset including results from cows with only one bacterial diagnosis per sampling event was used, i.e., cows with more than one bacterial finding in different quarters at one sampling event were excluded. The bacterial finding was set as the cow-level diagnosis.

For example, a cow with *S. aureus* in one quarter and *S. chromogenes* in another quarter at a sampling event, was counted as both *S. aureus* and *S. chromogenes* according to definition 1, as only *S. aureus* according to definition 2, and excluded from the dataset in definition 3.

Sampling events with no bacterial growth from any of the tested quarters were classified as negative (cow-level) in all three definitions. Samples reported as contaminated because > 2 different colony types were observed in at least one quarter, with no bacterial diagnoses on the remaining quarters were given a cow-level diagnosis of “contaminated”. IMI caused by other pathogens than the abovementioned were classified as “other”.

### Descriptive data analyses

The bacterial culture results were first summarized at quarter-level and the Chi square test was used to assess differences in distributions of each pathogen between front quarters and hind quarters. At cow-level, we summarized the data according to the three definitions defined above.

Results from staphylococcal penicillin resistance testing were described for all isolates tested, which means that up to four staphylococcal isolates could be tested per cow per sampling event, without considering clustering on cow-level.

Results from qPCR-analyses were summarized and the proportions of the four udder pathogens detected by the qPCR (*S. aureus, S. dysgalactiae, S. uberis, S. agalactiae*) were compared with bacterial culture results submitted with the following reasons for sampling “control before dry-off” and “*S. agalactiae*-control”.

### Analyses of statistical associations between bacterial culture findings and milking systems

Data were analysed in R (version 4.0.2) [12] and Stata (Release 14.2, Stata Corp. LLC, 2015). Results from bacterial culture were merged with herd-data including barn type (freestall vs. tiestall), milking system (pipeline, parlour, including carousel, or AMS), average milk yield, and herd size (number of cows). To correct for different sampling frequencies between herds, the proportion of sampled cows in the herd was calculated by dividing the total number of milk samples received from the herd by the number of cows in the herd, and then dividing this by two years. After plotting the distribution of samples per herd, herds with more than two samples per cow per year were considered non-representative and these herds were excluded.

Nine mixed multivariable logistic regression models were built, one model for each of the major and minor udder pathogens described above. The cow-level diagnosis was the outcome, using “definition 1”, i.e., cows with the udder pathogen detected vs all others (1/0), and including all reasons for sampling. Milking system was the independent variable of primary interest. Barn type was completely correlated with milking system and therefore not evaluated in the models. Herd was included as random effect, and herd size (annual average of number of lactating cows), herd average milk yield and the proportion of sampled cows (as described above) were included as confounders.

Less than four percent of the farms (n = 162) had more than 70 cows. Since the herd size varies between the milking systems, with the majority of AMS-herds having more than 30 cows, the models were also run on a dataset including a subset of samples from herds with between 30 and 70 cows. Model residuals were plotted along all the different covariates and along the predictor to check for nonlinearity, heteroscedasticity, and quality of model fit. To evaluate the overall performance of the models we generated in-sample receiver operating characteristic (ROC) curves and computed the area under the ROC-curve (AUC) [13]. To assess the farm-level variance, AUC was computed for the models with and without farm as random effect.

## Results

### Summary of bacterial culture results

The data included 155,692 quarter-level bacterial culture results from the same number of samples, obtained at 39,888 sampling events, giving an average of 3.9 quarter milk samples per sampling event. Hereafter, we refer to the sampling event, meaning the set of quarter-milk samples obtained from a cow on the same day, when the quarter-level is not specified.

Sampling events were excluded for the following reasons i) the same diagnosis from the same cow at multiple sampling events (n = 1709), ii) control after a mastitis treatment (n = 1390), iii) from farms with a more than two samples per cow per year (n = 276), iv) samples with antimicrobial residues (n = 63), or v) collected less than four days since the previous sampling event (n = 19).

The final dataset included 36,431 sampling events with 143,307 quarter-level diagnoses (Additional file 1). The samples came from 4158 farms, with a range of 1–201 sampling events per farm in the two-year period, and 30,154 cows with a range of 1–5 samples per cow. The majority of the cows (96%) had 1–3 sampling events in the two-year study period.

The reason for sampling was recorded as “subclinical mastitis” for 25,679 sampling events (71%), “clinical mastitis” for 7598 sampling events (21%), and “other reasons” for 3135 sampling events (9%), the latter included udder health control related to sale/purchase of cows and follow up in *S. agalactiae*-positive herds.

The final dataset with bacterial culture results included milk samples from 59% of the Norwegian dairy farms (4158 of 7000), with a similar distribution of herd size and barn systems to the total population. The farms submitting milk samples had a higher frequency of mastitis treatments and a lower milk yield compared to the average in the NDHRS (Table 1).

**Table 1:** Herd data from herds with milk samples in 2019 and 2020 *versus* all herds in Norway

	Herds with milk samples				All herds (n = 7070)^a^
	All herds (n = 4132)	Pipeline (n = 2359)	Parlour (n = 507)	AMS (n = 1266)	
Herd size, mean (SD)^b^	28 (18)	18 (8)	28 (16)	46 (17)	29
Average milk yield, mean (SD)^c^	8262 (1347)	7887 (1323)	8056 (1247)	9050 (1057)	8647^f^
Mastitis treatments, mean (SD)^d^	0.23 (0.2)	0.28 (0.2)	0.22 (0.2)	0.14 (0.1)	0.14
BMSCC, mean (SD)^e^	118 (37)	112 (37)	122 (39)	127 (34)	121^f^
Number of samples	36,286	15,849	5404	15,033	

^a^Number of dairy herds in Norway in 2019

^b^Number of lactating cows

^c^305-day lactation, energy corrected milk

^d^Mastitis treatments per cow year

^e^Bulk milk somatic cell count, 12-month geometric mean, × 1000 cells/mL

^f^Includes herds with at least three milk recordings

The quarter-level distribution of udder pathogens is provided in Additional file 1. Among the most common bacterial findings, all udder pathogens were significantly (P < 0.05) more prevalent in hind quarters, except for *T. pyogenes* and *S. epidermidis,* for which there was no difference, and *C. bovis*, that was significantly (P < 0.05) more prevalent in front quarters (Additional file 1). The cow-level distributions of udder pathogens (all sampling reasons) according to the three definitions are provided in Table 2. Non-aureus staphylococci as a group (n = 14,094) was the most common bacterial finding, detected in 39% of the sampling events according to definition 1 (Table 2, sum of NAS).

**Table 2:** Distribution of bacterial culture results in bovine milk samples in Norway (n = 36,431) analysed years 2019–2020

	Definition of cow-level diagnosis					
Bacterial finding	Definition 1^a^		Definition 2^b^		Definition 3^c^	
	n	%	n	%	n	%
Negative^d^	7743	21.2	7743	21.3	7743	30.1
Major pathogens						
* Staphylococcus aureus*	8918	24.5	7750	21.3	5028	19.6
* Streptococcus dysgalactiae*	4827	13.3	3618	9.9	2105	8.2
* Streptococcus uberis*	3269	9.0	2365	6.5	1411	5.5
* Escherichia coli*	1859	5.1	1493	4.1	936	3.6
* Trueperella pyogenes*	758	2.1	503	1.4	346	1.4
* Streptococcus agalactiae*	311	0.9	311	0.9	162	0.6
Mixed major^e^	–	–	2244	6.2	–	–
Minor pathogens						
* Staphylococcus epidermidis*	3630	10.0	1790	4.9	1327	5.2
* Staphylococcus chromogenes*	2180	6.0	1022	2.8	766	3.0
* Staphylococcus simulans*	1697	4.7	704	1.9	508	2.0
* Staphylococcus haemolyticus*	670	1.8	277	0.8	195	0.8
Other non-aureus staphylococci^f^	5917	16.3	2647	7.3	2237	8.7
* Corynebacterium bovis*	3418	9.4	903	2.5	901	3.5
Mixed minor^g^	–	–	907	2.5	–	–
Other bacteria^h^	–	–	974	2.7	870	3.4
Contamination^i^	2264	6.2	1180	3.2	1163	4.5
Total	47,461	1305^j^	36,431	100	25,698	100

^a^Definition 1: Each quarter sample diagnosis was included independent of the other quarter samples, meaning that cows with two different diagnoses at the same sampling occasion contributed to the frequency of both udder pathogens

^b^Definition 2: Cows with at least one quarter with *S. aureus, S. dysgalactiae, S. uberis, E. coli*, *T. pyogenes* or *S. agalactiae* (“major” pathogens) were assigned this as cow-level diagnosis. Cows were given the cow-level diagnosis NAS, *S. epidermidis, S. chromogenes, S. simulans, S. haemolyticus* or *C. bovis* (“minor pathogens”) if they had no major udder pathogen at the same sampling event

^c^Definition 3: Cow-level diagnoses in a subset with only one bacterial finding per sampling event

^d^No growth in all sampled quarters

^e^Cows with more than one major udder pathogen at the same sampling event

^f^Non-aureus staphylococci other than *S. epidermidis, S. chromogenes, S. simulans, S. haemolyticus*

^g^Cows with more than one minor udder pathogens at the same sampling event

^h^Bacteria other than presented in the table

^i^Samples with at least one quarter yielding more than two different colony types and no other udder pathogens in other quarters

^j^Giving on average 1.3 bacterial diagnoses per cow per sampling event

**Fig. 2:**
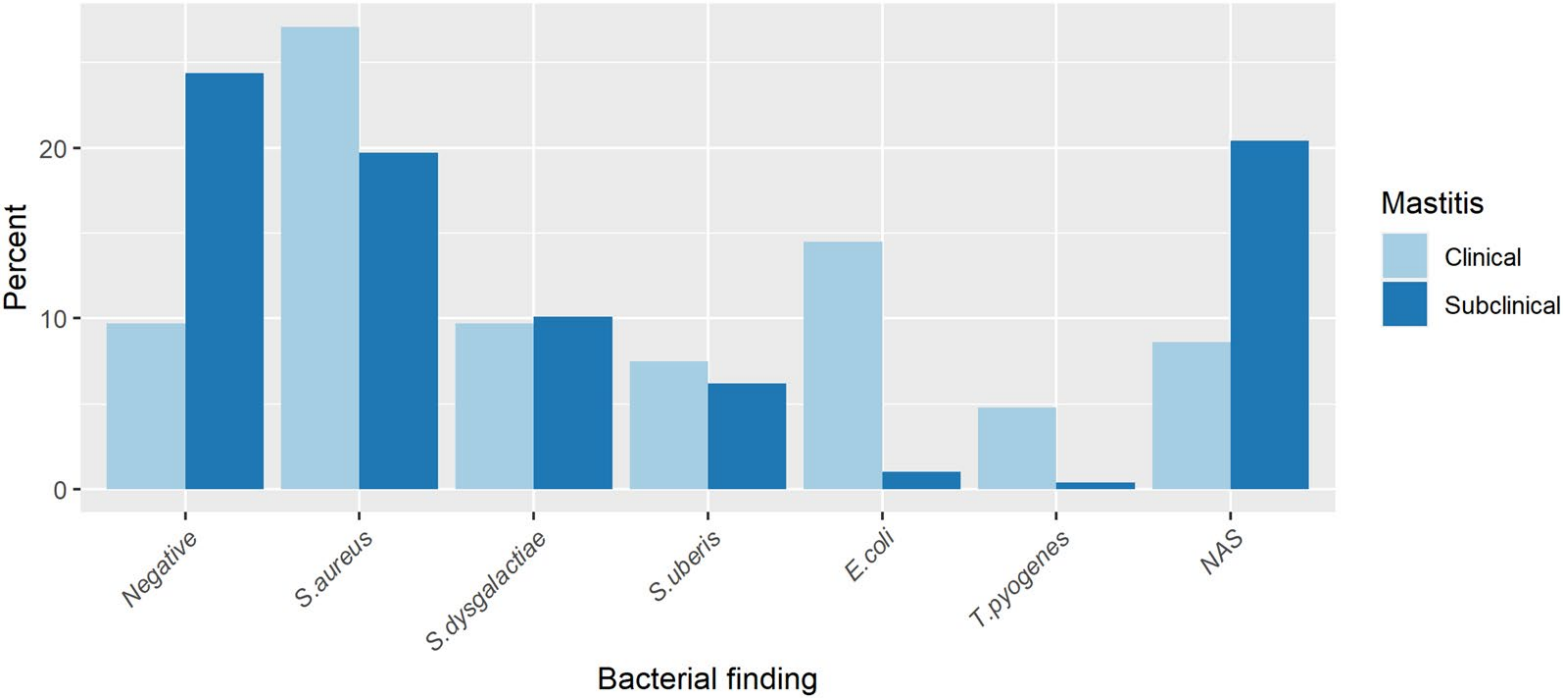
**Prevalence of selected udder pathogens in milk samples from clinical (n = 7598) and subclinical mastitis (n = 25,679).** Percentage of selected udder pathogens according to definition 1 (each quarter sample diagnosis was included independently of the other quarter samples, meaning that cows with two different diagnoses at the same sampling event contributed to the incidence of both udder pathogens). NAS = non-aureus staphylococci

Milk samples from clinical mastitis had a higher proportion of *S. aureus, S. uberis, E. coli* and *T. pyogenes* than samples from subclinical mastitis (Fig. 2), a finding valid for all three diagnosis definitions. NAS and no growth (negative) were more common in the samples from subclinical mastitis. The most common bacterial findings in samples from clinical mastitis were *S. aureus* (27.1%), *E. coli* (14.5%), and *S. dysgalactiae* (9.7%) (Fig. 2).

A total of 12,828 staphylococcal isolates were tested by the cloverleaf assay (Table 3). The proportion of *S. aureus* isolates with betalactamase-production was low (1.6–2.6%). Of the 2153 NAS tested for betalactamase-production, 740 (34%) were resistant to benzylpenicillin, with the proportion ranging from 1.9% (*S. simulans*) to 62.1% (*S. haemolyticus*).

**Table 3:** Resistance to benzylpenicillin in staphylococcal isolates from milk samples in Norway, 2019 and 2020

	All samples, n (%)		Clinical mastitis, n (%)	
Staphylococcus	Sensitive	Resistant	Sensitive	Resistant
*Staphylococcus aureus*	10,405 (97.5)	270 (2.5)	3114 (98.4)	52 (1.6)
*Staphylococcus epidermidis*	268 (50.5)	263 (49.5)	183 (51.0)	176 (49.0)
*Staphylococcus chromogenes*	261 (78.9)	70 (21.1)	175 (78.1)	49 (21.9)
*Staphylococcus simulans*	266 (98.2)	5 (1.9)	204 (97.6)	5 (2.4)
*Staphylococcus haemolyticus*	39 (37.9)	64 (62.1)	26 (36.1)	46 (63.9)
Other NAS^a^	579 (63.1)	338 (36.9)	417 (62.9)	246 (37.1)

^a^Non-aureus staphylococci other than *S. epidermidis, S. chromogenes, S. simulans, S. haemolyticus*

### Summary of qPCR results

Altogether 13,560 composite cow samples from 970 farms were analysed by the Mastit 4 M4A qPCR. Among these farms, 34% (n = 330) had submitted samples for qPCR-analysis only, whilst the remaining farms were also included in the data set describing bacterial culture results. The average herd size among farms submitting samples for qPCR-analysis was 37 cows (Standard deviation, SD 20). Seventy-five percent of the milk samples analysed by qPCR came from herds with AMS, whilst 17% and 7% were from tiestall and freestalls with milking parlour, respectively. Except for *S. agalactiae*, the distribution of the udder pathogens included in the Mastit 4 M4A qPCR was similar to the samples submitted with the same sampling reasons for bacterial culture (Table 4).

**Table 4:** Results from analysis of milk samples by qPCR and bacterial culture^a^

Bacterial finding	qPCR, n (%)	Bacterial culture, n (%)
Negative/other bacteria	8146 (60.1)	7153 (69,4)
*Staphylococcus aureus*	1865 (13.8)	1628 (15.8)
*Streptococcus dysgalactiae*	1,275 (9.4)	929 (9.0)
*Streptococcus uberis*	808 (6.0)	535 (5.2)
*Streptococcus agalactiae*	249 (9.0)	62 (0.6)
Total	13,560 (100)	10,307 (100)

^a^Samples from 970 (qPCR) and 1,489 (bacterial culture) dairy herds submitted to the TINE mastitis laboratory in Norway in 2019 and 2020 with reason for sampling i) control at dry-off (cows with somatic cell count geometric mean > 100,000 cells/mL the three milk recordings before dry-off, and ii) control of group B streptococci (*Streptococcus agalactiae*)

### Associations between bacterial culture results and milking systems

**Fig. 3:**
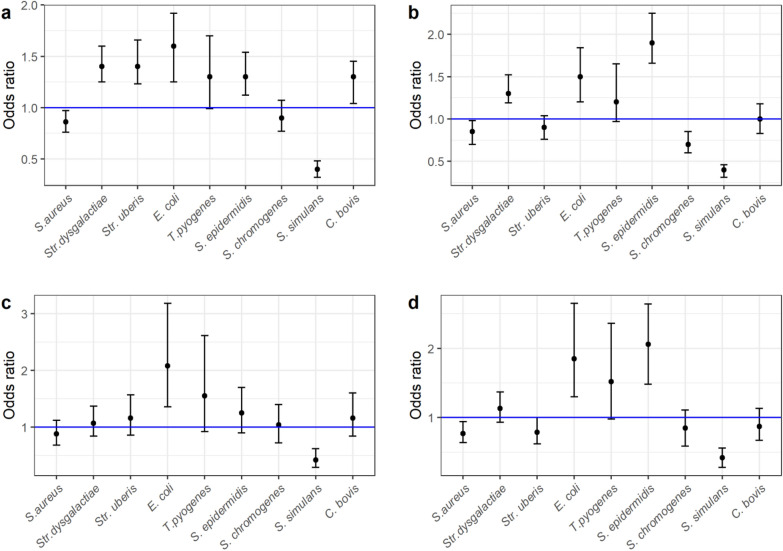
Coefficient plots for the association between bacterial finding (0/1) and milking system^1^. ^1^Odds ratio (95% confidence interval) for the association between cow-level bacterial finding (0/1) and milking system in logistic regression models including all observations (n = 36,286 milk samples from 4132 farms). Herd was included as random effect, and herd size and herd average milk yield was included as confounders. **a** Association between parlour milking (freestall) compared to tiestall pipeline **b** automatic milking systems (AMS) milking (freestall) compared to tiestall pipeline. Figure **c** and **d** provide the estimates for the same models on a subset of samples from farms with 30-70 cows (n = 18,119 milk samples from 1371 farms) for parlour milking compared to tiestall pipeline and AMS compared to tiestall pipeline, respectively

Data from 26 farms (126 samples) were excluded due to missing values. Thus, the data used in the multivariable mixed models included 36,305 sampling events and 4132 farms (Additional file 2). The models including all herds (n = 4132) showed that *S. aureus* was associated with tiestalls and pipeline milking, whilst *S. dysgalactiae* and *E. coli* were associated with freestall milking system (parlour and AMS). *S. epidermidis* was associated with AMS milking (Fig. 3a and b). The subset of data from farms with 30–70 cows (n = 1371 herds) showed an association only for *E. coli* with freestalls, *S. epidermidis* with freestall AMS milking, whilst *S. aureus* and *S. simulans* were associated with tiestall housing (Fig. 3c and d).

Adding herd as random effect increased the AUC for each of the udder pathogen models by 0.16–0.3 (Additional file 2). For *S. dysgalactiae, S. uberis*, *S. chromogenes* and *C. bovis,* a low proportion (AUC < 0.6) was explained by the fixed effects, whilst the AUC increased to > 0.75 by adding the random effect.

## Discussion

This study presents results from the routine diagnostics of milk samples analysed by bacterial culture (n = 36,431) and the Mastit 4 M4A qPCR (n = 13,560), retrieved from the NDHRS. Understanding how routines for sampling and diagnostic interpretations may affect results and the apparent distribution of udder pathogens in different types of farms is fundamental when informing strategies for the national udder health work. More than 4,000 farms, constituting approximately 60% of all Norwegian bovine dairy farms, were represented with samples in the dataset. These were considered representative of Norwegian bovine dairy farms with respect to geographical distribution, the number of animals, milking system, herd size, and housing systems. Samples analysed by qPCR more often came from larger than average dairy herds milked in AMS.

A limited number of studies have investigated the national prevalence of udder pathogens in different countries, and in the few studies available, the sampling, inclusion criteria and diagnostic methods have differed. Some studies have utilized random sampling [14, 15], or have only included affected quarters [16–18]. Others have utilized qPCR as the main diagnostic tool [19]. The prevalence of udder pathogens presented in the different studies is therefore not necessarily comparable.

The recommended Norwegian sampling strategy, to always sample all four quarters, enables comparison of clinically unaffected and affected quarters and helps inform the level of sample contamination. The idea is that it can support interpretation, but in fact it may also complicate it. Clearly, it is important to carefully consider the relevance of bacterial findings from diseased quarters in light of findings from quarters with no inflammatory response when interpreting results. Another relevant consideration is to evaluate the quality of the samples with respect to contaminant growth. Both major and minor udder pathogens can colonise or be present on teat skin and the teat canal and are likely contaminants of quarter milk samples. When information regarding quarter SCC, clinical signs, and sample contamination are used in combination with bacterial results to make decisions at cow level, the sampling strategy used in Norway can facilitate interpretation by providing control samples from each cow. However, without utilizing the mentioned metadata, the sampling strategy could potentially lead to unnecessary antimicrobial treatments and overestimation of the prevalence of IMI. Since information regarding clinical signs and somatic cell count at quarter level is not registered in NDHRS, it was not available in this study. Instead, different definitions of cow-level diagnoses were applied in the analyses in an attempt to decipher possible incidental bacterial findings, for example teat canal colonisations. When all bacterial findings were counted (definition 1), the prevalence of the udder pathogens defined as “minor” was relatively high. Although it is controversial to rank some udder pathogens to be more important than others (definition 2), the prevalence of the udder pathogens defined as “minor” using definition 2 showed a similar distribution to the subset of cows with only one bacterial diagnosis at one sampling event (definition 3), which may serve as a control group. This suggests that a considerable proportion of the bacterial findings in our dataset were incidental, and possibly did not reflect true intramammary infections.

The diagnostic criteria used in bacteriological analyses in the laboratory to define a quarter as infected, influence results and the interpretation of udder pathogen distribution. For example, the marked increase of NAS in Norway observed after 2005 (Fig. 1) may be explained by an alteration of the diagnostic criteria in the culture methodology in 2006, when the cut-off for defining an IMI was reduced from “rich pure growth” of a single colony type to “five colony forming units” of the same colony type. Differences in SCC cut-offs for diagnosing subclinical mastitis in a cow, and hence the recommendation for farmers regarding when to submit a milk sample, can also affect the apparent distribution of udder pathogens. A considerable proportion of cows recommended to test at dry-off in Norway, with a composite cow geometric mean of 100,000 cells/mL [3, 20], would not be tested in countries that apply a limit of 200,000 cells/mL [10, 21, 22].

There are also other possible explanations for altered reported prevalence of some udder pathogens in Norway over the past decades, including the increase of IMI caused by streptococci (Fig. 1), which may be related to changes in management during the same period. Norwegian dairy farms have undergone a process of modernization with a transition from smaller tiestall housing to larger modern dairy farms with freestall housing and often AMS milking. Our statistical models indicated an association between some udder pathogens and milking system or barn type. For many of the models, the association was not evident when analysing a subset of observations from herds with 30–70 cows, indicating that the herd size had a strong influence on the results. This is probably not only a direct effect of herd size, but rather related to a difference in management in small herds (< 30 cows), including regimens for milk sampling and mastitis treatment.

This study confirms that *S. aureus* remains the dominant udder pathogen of dairy cattle in Norway, as was also reported in 2006 [14]. An observed reduction in the prevalence of *S. aureus* IMI in dairy cows in many countries has been attributed to the implementation of the five-point plan and later the 10-point plan [23]. Some of these measures are less consistently followed in Norway. Routine use of post milking teat disinfection is currently not a general recommendation, and the use of selective dry cow therapy is preferred over blanket dry cow therapy [24]. Due to climatic conditions, the Nordic countries also have a relatively long housing period for the animals, which could contribute to transmission of contagious udder pathogens like *S. aureus* [25]. Furthermore, tiestalls are still common in the Nordic countries, a housing system shown to be a risk factor for *S. aureus* IMI in a Swedish study [18]. This was supported in the current study, where we found an association between *S. aureus* IMI and tiestall housing. However, when only farms with 30–70 cows were included in the models, the effect was non-significant for freestall parlour compared to tiestall pipeline milking, or less evident for AMS compared to pipeline milking. This could indicate that the association between *S. aureus* and tiestall housing is partly attributed to factors related to herd size, since the tiestall-herds are generally smaller. For example, farmers of smaller herds may be more reluctant to cull chronically infected cows.

In the data analyses using definition 1, a NAS was identified in 39% of the sampling events (Table 2, sum of all NAS); making NAS the most frequently identified udder pathogen in Norway. This is in agreement with a Canadian study [26], and studies from other European countries [15, 27]. NAS is a heterogenous group of bacteria, with different effects on the udder, on milk quality, and with different epidemiology [28–30]. The species-specific detection of NAS by MALDI TOF allowed us to demonstrate the marked differences of associations of NAS in different housing and milking systems. For example, *S. simulans*, like *S. aureus,* was associated with tiestalls. In contrast, *S. epidermidis* was associated with freestall herds (Fig. 3). *S. epidermidis* was the dominant NAS in our study, which is in agreement with results from Sweden [31]. In contrast, *S. chromogenes* is the most commonly detected NAS in bovine milk samples in most other countries [28, 30, 32]. Factors underlying the success of certain NAS in association with the bovine udder are still unexplored. The Nordic countries have a relatively high proportion of milk produced in AMS, which was associated with *S. epidermidis* in this study. In Norway, milking in AMS is also associated with larger herd size and higher production, which were included in the models as confounders.

Identification of NAS at species-level is one advantage of bacterial culture over the current commercial PCR methods and is one of the reasons why culture is still preferred in Norway. Another reason includes the fact that the repertoire of bacterial species detected is greater by culture compared to routinely applied qPCR methods which are limited to a selected panel of pathogens. Finally, bacterial culture enables AMR testing of isolates. The TINE mastitis laboratory charges the same cost for analyses of milk by culturing and qPCR to avoid farmers choosing qPCR based on price. Our results show that qPCR-analysis is primarily used by farms with AMS, possibly due to the opportunity to utilize samples already automatically collected for milk recordings. A common concern regarding these non-aseptically and automatically obtained samples is a higher rate of contamination and carry-over compared to aseptically obtained samples. This is, therefore, not a recommended sampling technique in Norway, but it is probably used to some extent. Although our data did not include results from the same samples analysed by the two different methods, it was interesting to observe that the prevalence of *S. aureus*, *S. uberis* and *S. dysgalactiae* was very similar in samples collected with the same reason for sampling.

It was not a surprise that the proportion of betalactamase producing *S. aureus* was very low in this study. This proportion has been stable over the past two decades, ranging from 3.5% to 1.5% of the isolates [33]. Reduction of antimicrobial usage has been an important goal in the Norwegian livestock industry, and in the dairy industry, antimicrobial treatments per cow-year have been reduced from 35 to 16% between 1996 and 2011 [34]. The national udder health work is believed to have contributed to this development.

Among the NAS isolates tested by the cloverleaf method, obtained mainly from clinical mastitis, the proportion of isolates resistant to benzylpenicillin was relatively high for *S. epidermidis* (50%) and *S. haemolyticus* (62%), and low for *S. simulans* (2%), but similar to reports by others [31, 35, 36]. It could be speculated that a higher occurrence of resistance to benzylpenicillin in *S. epidermidis* contributes to its dominance in NAS-IMI. However, this seems unlikely given that the treatment rate for clinical mastitis is 14% in Norway [6], which is relatively low. Furthermore, this has not been observed in other countries with higher antibiotic usage, where *S. chromogenes* dominates despite being mostly sensitive to benzylpenicillin [32, 37]. This difference in AMR among NAS further underlines the importance of detection of NAS at species-level to determine the AMR for each species.

## Conclusions

Milk sample bacteriology results extracted from a database covering a national population of dairy cows may be used to follow trends in the national distribution of udder pathogens and to identify risk factors. With the ongoing structural changes of the milk production in industrialized countries, updated knowledge of the prevalence of udder pathogens is essential. This study also shows that sampling strategies, herd size, and the applied diagnostic criteria influence the results significantly. This type of data should therefore be used with care when inferring prevalence and pathogen-specific risk factors.

## Supplementary Information

**Additional file 1.** Results from quarter milk samples (n = 143,307) analysed by bacterial culture in Norway, 2019–2020

**Additional file 2.** Odds ratio (OR) and 95% confidence interval (CI) estimates from nine mixed multivariable logistic regression models, with the cow-level diagnosis as outcome (cows with the udder pathogen detected vs all others (1/0)). The results are based on results from 36,305 sampling events (each with up to four quarter milk samples per cow) from 30,154 cows in 4,132 Norwegian dairy farms, retrieved from the Norwegian Dairy Herd Recording System and including years 2019 and 2020. The explanatory variable of main interest was the barn- and milking system. Herd size (number of lactating cows), average annual herd milk yield and the proportion of cows sampled were included as confounders. Herd was included as random effect. *indicates a significant association with the outcome (P < 0.05). The area under the ROC-curve (AUC) for each model is calculated for the model with and without herd as random effect

## Data Availability

The datasets used during the current study are available from the corresponding author on reasonable request.
