# Supplementary figures and images for: Prevalence of udder pathogens in milk samples from Norwegian dairy cows recorded in a national database in 2019 and 2020
Source: Acta Vet Scand. 2023 Jun 1;65:19. doi: 10.1186/s13028-023-00681-2 (PMC10234032; doi:10.1186/s13028-023-00681-2)

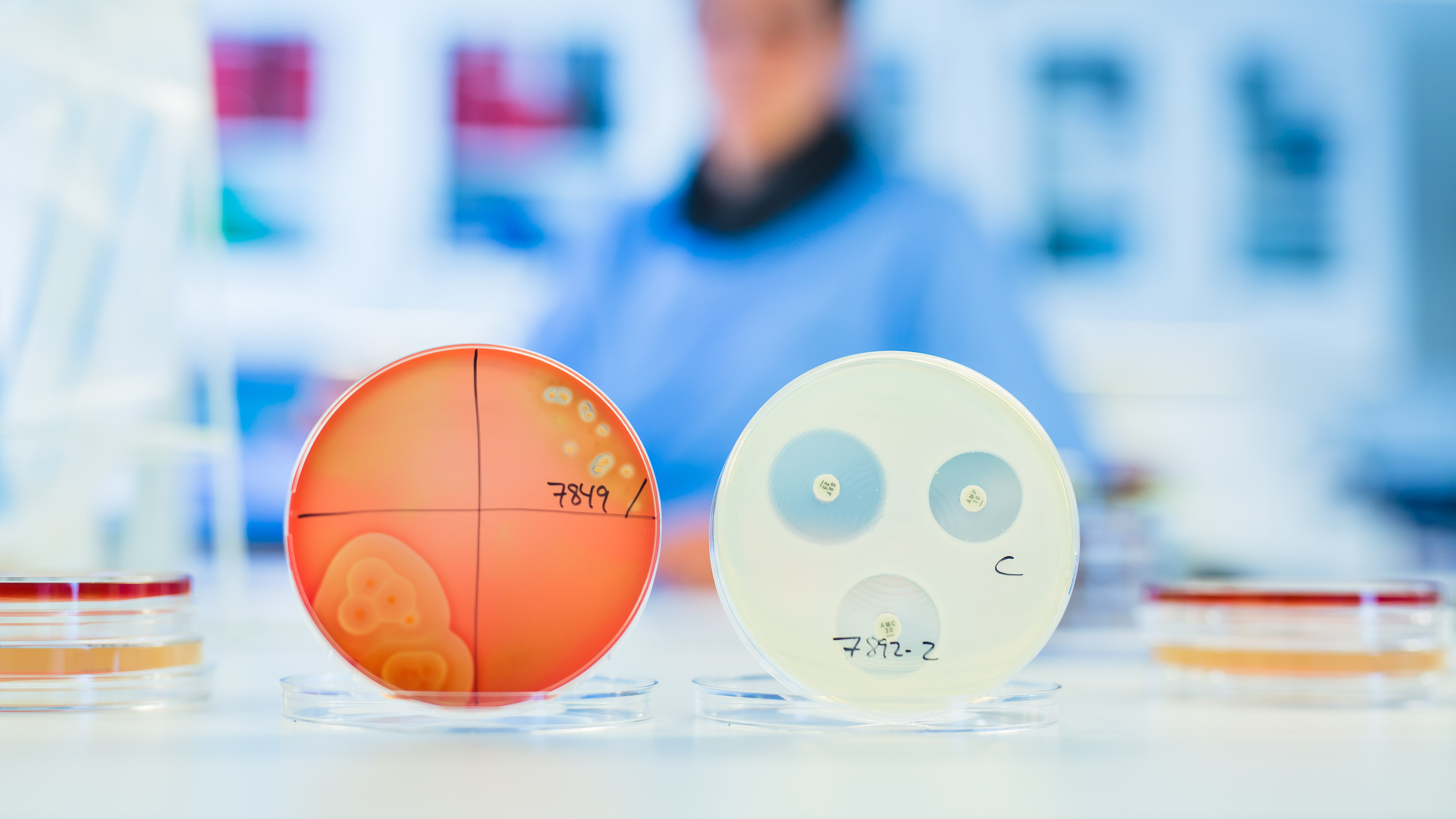

Supplement: Supplementary file 1 — Additional file 1. Results from quarter milk samples (n = 143,307) analysed by bacterial culture in Norway, 2019–2020 [file 13028_2023_681_MOESM1_ESM.jpg]
