# Supplementary material for: Prevalence of udder pathogens in milk samples from Norwegian dairy cows recorded in a national database in 2019 and 2020
Source: Acta Vet Scand. 2023 Jun 1;65:19. doi: 10.1186/s13028-023-00681-2 (PMC10234032; doi:10.1186/s13028-023-00681-2)
Supplement: Supplementary file 2 — Additional file 2. Odds ratio (OR) and 95% confidence interval (CI) estimates from nine mixed multivariable logistic regression models, with the cow-level diagnosis as outcome (cows with the udder pathogen detected vs all others (1/0)). The results are based on results from 36,305 sampling events (each with up to four quarter milk samples per cow) from 30,154 cows in 4,132 Norwegian dairy farms, retrieved from the Norwegian Dairy Herd Recording System and including years 2019 and 2020. The explanatory variable of main interest was the barn- and milking system. Herd size (number of lactating cows), average annual herd milk yield and the proportion of cows sampled were included as confounders. Herd was included as random effect. *indicates a significant association with the outcome (P < 0.05). The area under the ROC-curve (AUC) for each model is calculated for the model with and without herd as random effect [file 13028_2023_681_MOESM2_ESM.pdf]

Additional file 2: Odds ratio (OR) and 95% confidence interval (CI) estimates from nine mixed multivariable logistic regression models, with the cow-level diagnosis as outcome (cows with the udder pathogen detected vs all others (1/0)). The results are based on results from 36,305 sampling events (each with four quarter milk samples per cow) from 30,154 cows in 4132 Norwegian dairy farms, retrieved from the Norwegian Dairy Herd Recording System and including years 2019 and 2020. The explanatory variable of main interest was the barn and milking system. Herd size (number of lactating cows), average annual herd milk yield and the proportion of cows sampled were included as confounders. Herd was included as random effect. OR=Odds ratio. Ref=Referent category. *indicates a significant association with the outcome (p <0.05). The area under the ROC-curve (AUC) for each model is calculated for the model with and without herd as random effect.

	S. aureus n=8918		*S. dysgalactiae* n=4827		*S. uberis* n=3269		*E. coli* n=*1859*		*T. pyogenes* n=758		*S. epidermidis* n=3630		*S. chromogenes* n=2175		*S. simulans* n=1691		*C. bovis* n=3409	
	OR	CI	OR	CI	OR	CI	OR	CI	OR	CI	OR	CI	OR	CI	OR	CI	OR	CI
Barn/milking system																		
Tiestall-pipeline	Ref		Ref		Ref		Ref		Ref		Ref		Ref		Ref		Ref	
Freestall-parlour	0.9*	0.76-0.97	1.4*	1.25-1.60	1.4*	1.23-1.66	1.6*	1.25-1.92	1.3	0.99-1.7	1.3*	1.12-1.54	0.9	0.77-1.07	0.4*	0.32-0.48	1.3*	1.04-1.45
Freestall-AMS	0.8*	0.70-0.98	1.3*	1.19-1.52	0.9	0.76-1.04	1.5*	1.20-1.84	1.2	0.97-1.65	1.9*	1.66-2.25	0.7*	0.60-0.85	0.4*	0.31-0.46	1.0	0.83-1.18
Herd size (per 10 cows)	0.9*	0.84-0.89	1.1*	1.06-1.12	1.0	0.96-1.03	1.1*	1.06-1.17	1.1 *	1.04-1.16	1.0	0.96-1.03	1.0	0.98-1.05	1.0	0.95-1.03	1.0*	1.0-1.09
Milk yield (per 500 kg)	1.0	1.0-1.03	1.0	0.99-1.02	0.9*	0.95-0.99	1.0	0.96-1.02	1.0	1.04-1.16	1.1*	1.03-1.07	1.0	0.99-1.04	1.03*	1.01-1.06	0.9*	0.95-0.99
Proportion sampled	0.6*	0.47-0.65	0.6*	0.47-0.66	0.6*	0.49-0.75	0.1*	0.11-0.21	0.2 *	0.13-0.29	1.3*	1.1-1.65	1.3*	1.08-1.64	1.0	0.81-1.28	1.1	0.89-1.44
AUC with random effect	0.76		0.75		0.82		0.88		0.9 2		0.79		0.82		0.81		0.83	
AUC without random effect	0.60		0.59		0.56		0.64		0.6 3		0.60		0.54		0.62		0.53	
